# Characterization of the complete chloroplast genome of *Angelica dahurica* (Apiaceae) as an herb in China

**DOI:** 10.1080/23802359.2020.1714493

**Published:** 2020-01-16

**Authors:** Rui Zhang, Baohai Xu, Tianyi Cao

**Affiliations:** a Beijing Jishuitan Hospital, Beijing, P. R. China;; b Department of Orthopaedics, Zhejiang Integrated Traditional Chinese and Western Medicine Hospital, Hangzhou, P. R. China

**Keywords:** *Angelica dahurica*, Apiaceae, chloroplast genome, genome, phylogenetic relationships

## Abstract

*Angelica dahurica* is an upright perennial herb that is becoming more and more fashionable in the garden in the world. The complete chloroplast genome of *A. dahurica* was annotated and studied in this paper. It was a circular molecular genome with a size of 146,918 bp in length, which was composed of four distinct regions such as a large single-copy region of 93,605 bp, a small single-copy region of 17,669 bp and a pair of IR regions of 17,822 bp. We annotated and found comprised 129 genes, including 85 protein-coding genes (PCGs), 36 transfer RNA genes (tRNAs) and eight ribosome RNA genes (rRNAs). The overall nucleotide composition is A of 30.8%, T of 31.7%, C of 19.1% and G of 18.4%, with a total A + T content of the chloroplast genome 62.5% and G + C of 37.5%. Phylogenetic analysis with the reported chloroplast genomes revealed that *A. dahurica* is most closely related to *Angelica gigas* in the phylogenetic relationships.

*Angelica dahurica* is a widely-cultivated perennial herb that found naturally in moist grassland and streamside. It grew mainly in China, Korea, Japan, Russia and other Southeast Asian countries, in which the cultivated herb is mainly from central and eastern regions from China. It has been used for centuries and is recorded to have been in use in Ancient China as early as 400 BC, which known is China is reputed to be an herb that will purge the body of any negative influences. The roots of *A. dahurica* called Bai-Zhi in traditional Chinese medicine that classified as a sweat-inducing drug able to counter harmful external influences on the skin, such as cold, heat, dampness and dryness (Lechner et al. 2004). Now, it has no much the genome information of *A. dahurica*, which also has no molecular biology data information. So, in this study, we annotated the complete chloroplast genome of *A. dahurica* and researched phylogenetic relationship with other plants species, which provides more databases resource and herbal medicines resource

Using the Plant Tissues Genomic DNA Extraction Kit (TIANGEN, BJ and CN) method, the total genomic DNA was isolated from the fresh of *A. dahurica* and collected from herb market near Zhejiang Chinese Medical University that located at Hangzhou, Zhejiang, China (30.09 N, 119.89E). The chloroplast genome DNA was stored in Zhejiang Chinese Medical University (No. SCMC-ZJU-TCM-07). Quality and adapters control was performed and removed low-quality reads and adapters using the NGS QC Toolkit software (Patel and Jain 2012). The chloroplast genome of *A. dahurica* was assembled and annotated using the MitoZ software (Meng et al. 2019). The complete physical map of *A. dahurica* chloroplast genome was generated using the OGDRAW version 1.3.1 (Greiner et al. 2019). The annotated chloroplast genome sequence was from the GenBank accession No. KT963037.

The complete chloroplast genome of *A. dahurica* was a circular molecular genome with a size of 146,918 bp in length, which was composed of four distinct regions such as a large single-copy region (LSC) of 93,605 bp, a small single-copy region (SSC) of 17,669 bp and a pair of inverted repeat regions (IRs) of 17,822 bp in each. We annotated and found the chloroplast genome comprised 129 genes, including 85 protein-coding genes (PCGs), 36 transfer RNA genes (tRNAs) and 8 ribosomal RNA genes (rRNAs). The overall nucleotide composition is 30.8% of A, 31.7% of T, 19.1% of C, and 18.4% of G, with a total A + T of 62.5% and G + C content of 37.5%.

In order to understand the phylogenetic relationship between *A. dahurica* and related species, the complete chloroplast genome sequences of 12 plant species were reconstructed trees using the Maximum-Likelihood (ML) methods. The phylogenetic tree was inferred phylogenetic using RaxML version 8.0 (Stamatakis 2014) with the most appropriate model. The phylogenetic tree was performed using MEGA X software (Kumar et al. 2018) by 2000 bootstrap replicates and edited using iTOL version 4.0 (https://itol.embl.de/) (Letunic and Bork 2019). As was expected, the phylogenetic tree (Figure 1) result shown that *A. dahurica* is closely grouped to *Angelica gigas* (NC_029393.1) in the phylogenetic relationship from the family Apiaceae.

**Figure 1.**
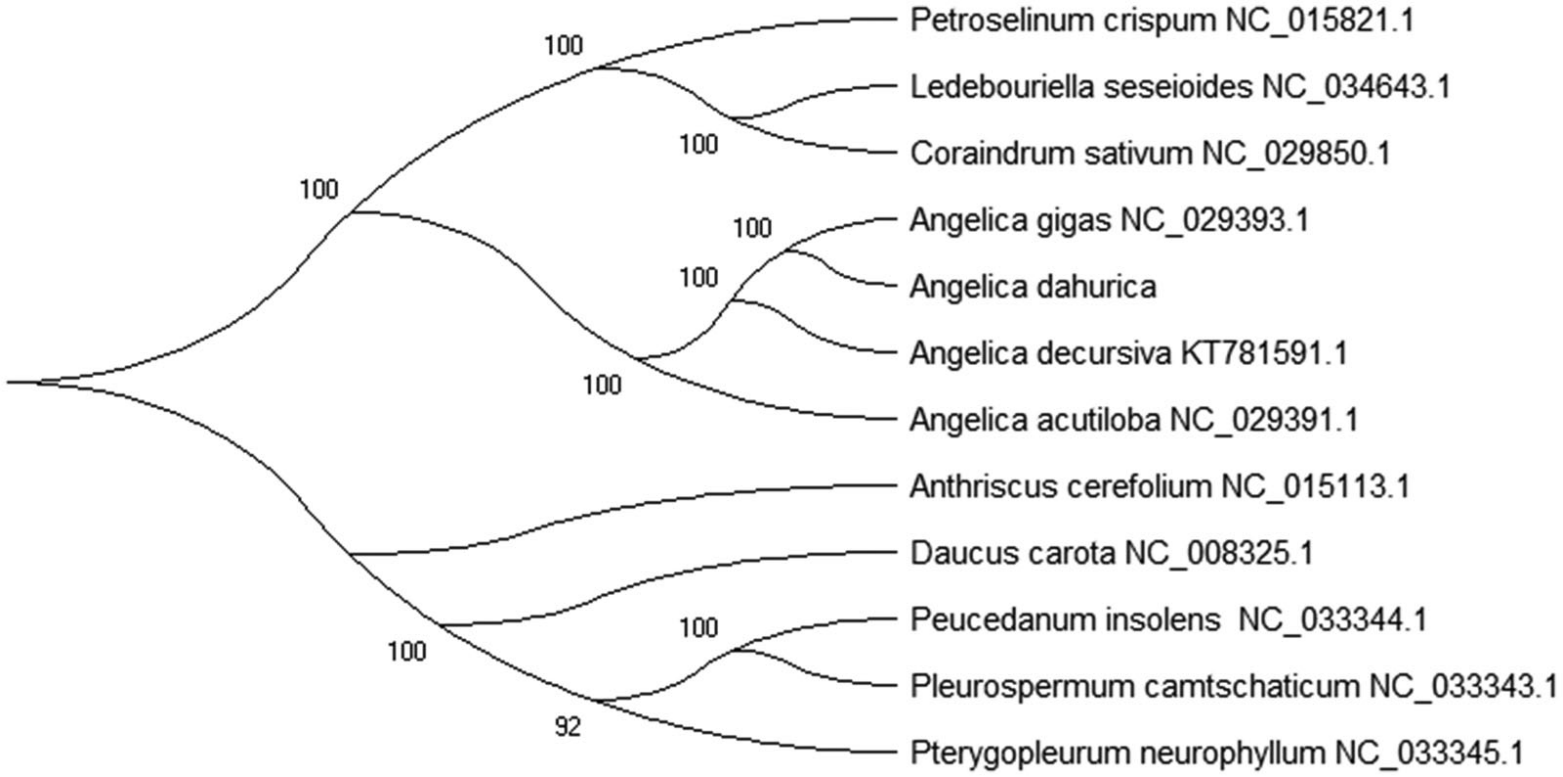
Maximum-Likelihood (ML) phylogenetic tree of *Angelica dahurica* with 12 species was constructed by chloroplast genome sequences. Numbers in the nodes are bootstrap values from 2000 replicates. The chloroplast genome sequences in this study have been deposited in the GenBank.
